# Carboxylesterase 1 Is Regulated by Hepatocyte Nuclear Factor 4α and Protects Against Alcohol- and MCD diet-induced Liver Injury

**DOI:** 10.1038/srep24277

**Published:** 2016-04-14

**Authors:** Jiesi Xu, Yang Xu, Yuanyuan Li, Kavita Jadhav, Min You, Liya Yin, Yanqiao Zhang

**Affiliations:** 1 Department of Integrative Medical Sciences, Northeast Ohio Medical University, Rootstown, OH 44272, USA; 2 Department of Pharmaceutical Sciences, Northeast Ohio Medical University, Rootstown, OH 44272, USA

## Abstract

The liver is a major organ that controls hepatic and systemic homeostasis. Dysregulation of liver metabolism may cause liver injury. Previous studies have demonstrated that carboxylesterase 1 (CES1) regulates hepatic triglyceride metabolism and protects against liver steatosis. In the present study, we investigated whether CES1 played a role in the development of alcoholic liver disease (ALD) and methionine and choline-deficient (MCD) diet-induced liver injury. Both hepatocyte nuclear factor 4α (HNF4α) and CES1 were markedly reduced in patients with alcoholic steatohepatitis. Alcohol repressed both HNF4α and CES1 expression in primary hepatocytes. HNF4α regulated CES1 expression by directly binding to the proximal promoter of CES1. Global inactivation of CES1 aggravated alcohol- or MCD diet-induced liver inflammation and liver injury, likely as a result of increased production of acetaldehyde and reactive oxygen species and mitochondrial dysfunctions. Knockdown of hepatic CES1 exacerbated ethanol-induced steatohepatitis. These data indicate that CES1 plays a crucial role in protection against alcohol- or MCD diet-induced liver injury.

Alcohol overconsumption may cause alcoholic liver disease (ALD), which encompasses alcoholic fatty liver disease (AFLD), alcoholic hepatitis (AH) and alcoholic cirrhosis. ALD is the major cause of liver disease in Western countries. Liver cirrhosis is the 12^th^ leading cause of death in the US, with a total of 36,427 deaths in 2013, 49.8% of which are related to alcohol^1^. Abstinence is a common and effective strategy for the treatment of ALD, but sustainable lifestyle changes are difficult for many patients to achieve. Pharmacological treatments, such as the use of alcohol dehydrogenase inhibitors and anti-craving drugs, do not achieve satisfactory results in patients with ALD^2^.

Significant advances have been made toward understanding the pathogenesis of ALD. Alcohol-induced increases in reactive oxygen species (ROS), lipid peroxidation (LPO), acetaldehyde, fatty acid ethyl ester (FAEE), lipopolysaccharide (LPS) and protein carbonyl, etc. are directly responsible for the development of ALD^3^,^4^,^5^,^6^,^7^. In addition, alcohol metabolism causes increased ratio of NADH/NAD^+^, which in turn suppresses the sirtuin 1 (SIRT1)-AMP-activated protein kinase (AMPK) axis, leading to induction of sterol regulatory element-binding protein 1 (SREBP-1) and lipogenesis and inhibition of fatty acid oxidation (FAO)^8^,^9^,^10^. As a result, alcohol overconsumption results in AFLD and AH.

Hepatic steatosis is an early hallmark of ALD and is a target for managing this disease. Carboxylesterase 1 (CES1) is a drug-metabolizing enzyme that is also capable of hydrolyzing triglycerides (TG) and cholesterol esters (CE)^11^,^12^,^13^. Over-expression of hepatic CES1 reduces TG accumulation through promoting lipolysis and FAO. In contrast, knockdown of hepatic CES1 increases lipid accumulation by inducing lipogenesis^11^,^14^,^15^. So far, the role of CES1 in the development of ALD or liver injury is completely unknown.

Hepatocyte nuclear factor 4α (HNF4α) is a nuclear hormone receptor that is constitutively active and regulates lipid, glucose, bile acid and drug metabolism. Loss of hepatic HNF4α causes fatty liver by inhibiting very low-density lipoprotein (VLDL) secretion^16^,^17^. HNF4α expression is markedly reduced in diabetes, obesity, non-alcoholic fatty liver disease (NAFLD) and high fat diet (HFD) feeding, likely resulting from increased free fatty acids (FFA), cholesterol and miR-34a expression. HNF4α controls the basal expression of many genes in the liver, but it is unclear whether HNF4α also regulates CES1 expression.

In the present study, we first investigated hepatic CES1 expression in patients with alcoholic steatohepatitis. We then investigated how alcohol downregulated hepatic CES1 expression and whether CES1 played a role in the development of ALD and non-alcoholic liver injury. Our data show that ethanol reduces hepatic CES1 expression likely through inhibition of HNF4α and that CES1 plays a crucial role in preventing ethanol- or methionine and choline-deficient (MCD) diet-induced liver injury.

## Results

### Alcohol reduces CES1 and HNF4α expression in patients with alcoholic steatohepatitis and in mice treated with alcohol

**Figure 1:**
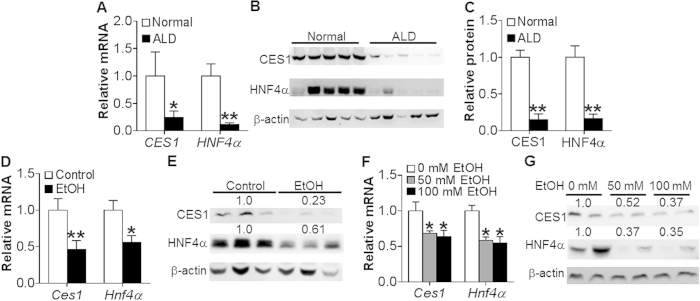
Alcohol inhibits CES1 and HNF4α expression in patients with alcoholic steatohepatitis and in mice. (**A**–**C**) Livers were collected from normal individuals or patients with alcoholic steatohepatitis (n = 7). mRNA levels were determined by qRT-PCR (**A**). Protein levels were determined by Western blotting (**B**) and then quantified (**C**). (**D**,**E**) C57BL/6 mice were fed a Lieber-DeCarli diet for 5 days, followed by fed a Lieber-DeCarli diet containing 5% ethanol or pair-fed a control diet for 10 days. On the 16^th^ day, mice were gavaged with a single dose of ethanol (5 g/kg) (n = 8). mRNA levels were then determined by qRT-PCR (**D**) and protein levels determined by Western blots (**E**). (**F**,**G**) Mouse primary hepatocytes were treated with 0, 50 or 100 mM ethanol for 24 h (n = 3). mRNA (**F**) and protein (**G**) levels were determined. *p < 0.05, **p < 0.01. Unpaired Student *t*-test was used for statistical analysis.

To investigate whether CES1 is associated with the development of ALD, we investigated the expression of CES1 in patients with alcoholic steatohepatitis. Hepatic *CES1 *mRNA level was reduced by 75% (Fig. 1A) and protein level decreased by ~85% (Fig. 1B,C). Interestingly, the nuclear receptor HNF4α was also markedly repressed by >84% in both mRNA (Fig. 1A) and protein (Fig. 1B,C) levels.

In mice chronically fed a Liber-DeCarli ethanol diet for 10 days plus binge ethanol (5 g/kg)^18^, hepatic *Ces1* and *Hnf4*α mRNA (Fig. 1D) and protein (Fig. 1E) levels were decreased. Consistent with the *in vivo* data, ethanol treatment repressed *Ces1* and *Hnf4*α mRNA (Fig. 1F) and protein (Fig. 1G) levels in primary hepatocytes. Thus, the data of Fig. 1 indicate that ethanol inhibits hepatic CES1 and HNF4α expression in both mice and humans.

### HNF4α regulates CES1 expression via binding to a DR-1 binding

**Figure 2:**
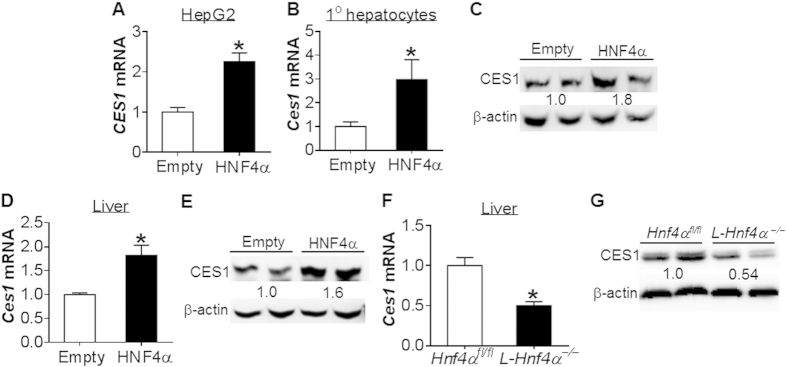
HNF4α regulates CES1 expression. (**A**–**C**) HepG2 cells (**A**) or mouse primary hepatocytes (**B**,**C**) were infected with adenoviruses expressing HNF4α (Ad-HNF4α; HNF4α) or control adenoviruses (Ad-empty; empty) (n = 3). After 48 h, *Ces1* mRNA (**A**,**B**) and protein (**C**) levels were determined. (**D**,**E**) C57BL/6 mice were injected with Ad-empty or Ad-HNF4α (n = 6). After 7 days, hepatic *Ces1* mRNA (**D**) and protein (**E**) levels were determined. (**F**,**G**) Hepatic *Ces1* mRNA (**F**) and protein (**G**) levels were determined in *Hnf4*α^*fl/fl*^ mice and *L-Hnf4*α^−/−^mice (n = 5). *p < 0.05. Unpaired Student *t*-test was used for statistical analysis.

The finding that alcohol inhibits both HNF4α and CES1 expression suggests that HNF4α may regulate CES1 expression. Over-expression of HNF4α induced *CES1* mRNA level by 2.3 fold in HepG2 cells (Fig. 2A). HNF4α over-expression also induced *Ces1* mRNA (Fig. 2B) and protein (Fig. 2C) levels in mouse primary hepatocytes. In line with the *in vitro* data, hepatic expression of HNF4α induced *Ces1* mRNA (Fig. 2D) and protein (Fig. 2E) levels in mice. In contrast, *Ces1* mRNA (Fig. 2F) and protein (Fig. 2G) levels were reduced by ~50% in liver-specific *Hnf4*α^−/−^ (*L-Hnf4*α^−/−^) mice. These data indicate that HNF4α regulates CES1 expression both *in vitro* and *in vivo*.

**Figure 3:**
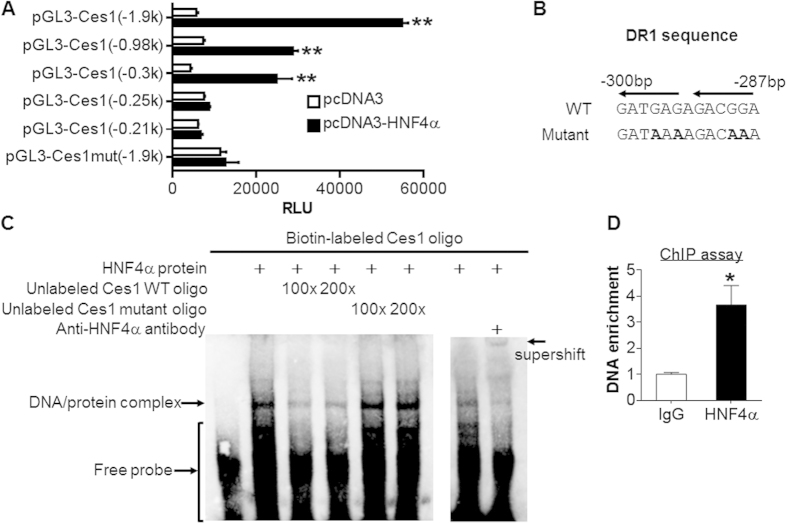
CES1 is a direct target of HNF4α. (**A**) Luciferase reporter assays were performed by co-transfecting HepG2 cells with pGL3-Ces1 luciferase-reporter constructs together with pcDNA3 or pcDNA3-HNF4α plasmids (n = 6). After 36 h, luciferase activities were determined and normalized to β-galactosidase activity. (**B**) Wild-type (WT) or mutant HNF4α response element in the *Ces1* gene promoter is shown in the top or bottom, respectively. (**C**) EMSA assays were performed using *in vitro* transcribed/translated HNF4α protein. Wild-type and mutant oligonucleotides were used in the competition assays (left panel). Supershift assays were performed in the presence of an HNF4α antibody (right panel). (**D**) Chromatin immunoprecipitation assays were performed using liver lysates and an HNF4α antibody. *p < 0.05, **p < 0.01. Unpaired Student *t*-test was used for statistical analysis.

Next, we examined how HNF4α regulates CES1 expression. Promoter-luciferase assays showed that HNF4α induced the luciferase activity by 10 fold, 4 fold and 6 fold in the −1.9 kb, −0.98 kb and −0.3 kb *Ces1* promoters, respectively, but not in the −0.25 kb *Ces1* promoter (Fig. 3A), suggesting that HNF4α may bind to the response element(s) located between −0.3 kb and −0.25 kb of the *Ces1* promoter. Indeed, there was a potential DR-1 element (direct repeat separated by one base pair) between 300 bp and 287 bp upstream of the transcription start site (Fig. 3B). Mutation of the DR-1 element abolished the induction of *Ces1* promoter activity by HNF4α (Fig. 3A). Electrophoretic mobility shift assays showed that HNF4α protein bound to the DR-1 element and this binding was inhibited by wild-type but not mutant DR-1 oligonucleotides (Fig. 3C, left panel). In addition, an HNF4α antibody was able to supershift the DNA/protein complex (Fig. 3C, right panel), indicating that HNF4α binds to the DR-1 element *in vitro*. Finally, chromatin immunoprecipitation assays showed that HNF4α protein bound to the *Ces1* promoter containing the DR-1 element in the liver (Fig. 3D). Collectively, the data of Figs 2 and 3 demonstrate that HNF4α regulates *Ces1* expression by binding to a DR-1 element located between 300 bp and 287 bp upstream of the transcription start site.

### Global inactivation of CES1 aggravates MCD diet-induced liver inflammation

**Figure 4:**
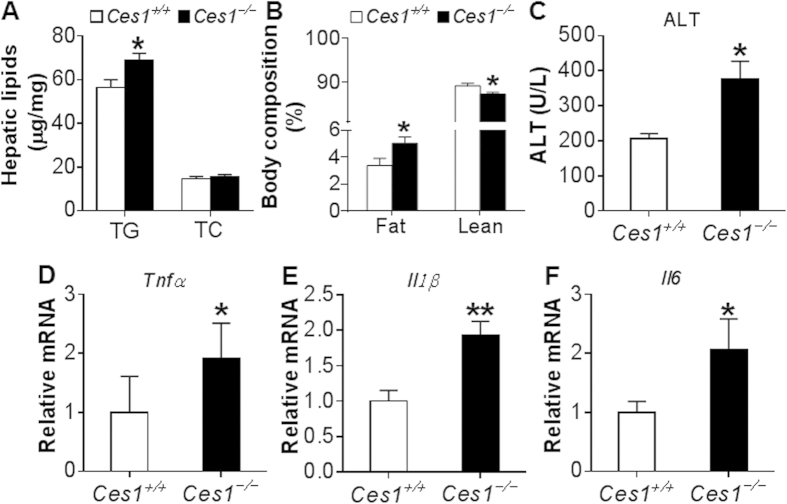
*Ces1*^−/−^ mice are susceptible to MCD diet-induced liver injury. (**A**–**F**) *Ces1*^+/+^ mice and *Ces1*^−/−^ mice were fed an MCD diet for 4 weeks. Hepatic triglycerides (TG) and total cholesterol (TC) (**A**), body fat composition (**B**), plasma ALT level (**C**), as well as hepatic mRNA levels of *Tnf*α (**D**), *Il1*β (**E**), and *Il6* (**F**) were determined. *p < 0.05, **p < 0.01. Unpaired Student *t*-test was used for statistical analysis.

Hepatic CES1 is known to regulate lipid and glucose metabolism^11^,^12^,^14^. However, the role of CES1 in liver inflammation or injury is unknown. We have therefore generated global *Ces1*^−/−^ mice, which did not express any *Ces1* mRNA or protein in the liver or other tissues (see Supplementary Fig. S1). When *Ces1*^+/+^ mice and *Ces1*^−/−^ mice were fed an MCD diet for 4 weeks, there was a 21% increase in hepatic TG level but hepatic cholesterol level was unchanged (Fig. 4A). *Ces1*^−/−^ mice had more fat accumulation than *Ces1*^+/+^ mice (5.03% vs. 3.37%) but reduced lean mass (Fig. 4B). *Ces1*^−/−^ mice also had increased plasma alanine aminotransferase (ALT) level (Fig. 4C) as well as hepatic mRNA levels of tumor necrosis factor α (*Tnf*α) (Fig. 4D), interleukin 1β (*Il1*β) (Fig. 4E), and interleukin 6 (*Il6*) (Fig. 4F). These data demonstrate that loss of CES1 aggravates MCD diet-induced liver inflammation and liver injury.

### Global inactivation of CES1 exacerbates alcohol-induced hepatitis

**Figure 5:**
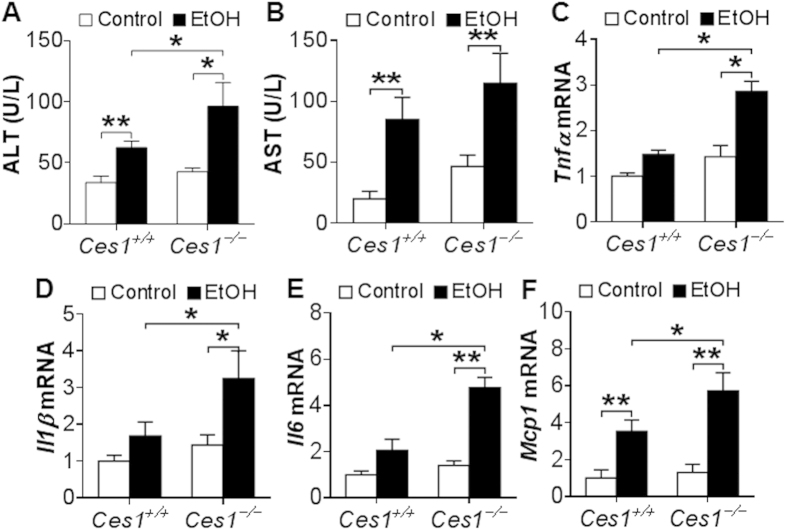
*Ces1*^−/−^ mice are susceptible to alcohol-induced liver inflammation. *Ces1*^+/+^ and *Ces1*^−/−^ mice were fed a Lieber-DeCarli control liquid diet for 5 days, followed by fed a Lieber-DeCarli ethanol diet or pair-fed a control diet for 10 days. On the 16^th^ day, a single dose of ethanol (3 g/kg) or isocaloric maltose dextrin was administered. Plasma levels of ALT (**A**) and AST (**B**) as well as hepatic mRNA levels of *Tnf*α (**C**), *Il1*β (**D**), *Il6* (**E**) and *Mcp-1* (**F**) were determined. *p < 0.05, **p < 0.01. Unpaired Student *t*-test was used for statistical analysis.

To test whether global inactivation of CES1 affects the development of ALD, *Ces1*^+/+^ mice and *Ces1*^−/−^ mice were fed a Lieber-DeCarbli diet containing 5% ethanol or pair-fed a control diet for 10 days followed by a single gavage of ethanol (3 g/kg) as described^18^. The chronic binge drinking did not affect their body weight (see Supplementary Fig. S2). Ethanol feeding significantly increased plasma levels of ALT (Fig. 5A), AST (Fig. 5B) and TG (see Supplementary Fig. S2) in both *Ces1*^+/+^ mice and *Ces1*^−/−^ mice, and such increases were further enhanced by *Ces1* inactivation (Fig. 5A,B and see Supplementary Fig. S2). In contrast, ethanol-induced increase in hepatic TG levels were comparable between *Ces1*^+/+^ mice and *Ces1*^−/−^ mice (see Supplementary Fig. S2). In addition, ethanol significantly induced hepatic mRNA levels of *Tnf*α (Fig. 5C), *Il1*β (Fig. 5D), *Il6* (Fig. 5E) and *Mcp1* (Fig. 5F) in *Ces1*^−/−^ mice, and the induction of *Tnf*α, *Il1*β and *Il6* was not significant in *Ces1*^+/+^ mice (Fig. 5C–E). Importantly, *Ces1* inactivation significantly potentiated ethanol-induced increase in *Tnf*α, *Il1*β, *Il6* and *Mcp1* mRNA levels in the liver (Fig. 5C–F). Thus, global loss of CES1 aggravates ethanol-induced liver injury.

### Global inactivation of CES1 aggravates ethanol-induced acetaldehyde and oxidative stress production and mitochondrial dysfunction

**Figure 6:**
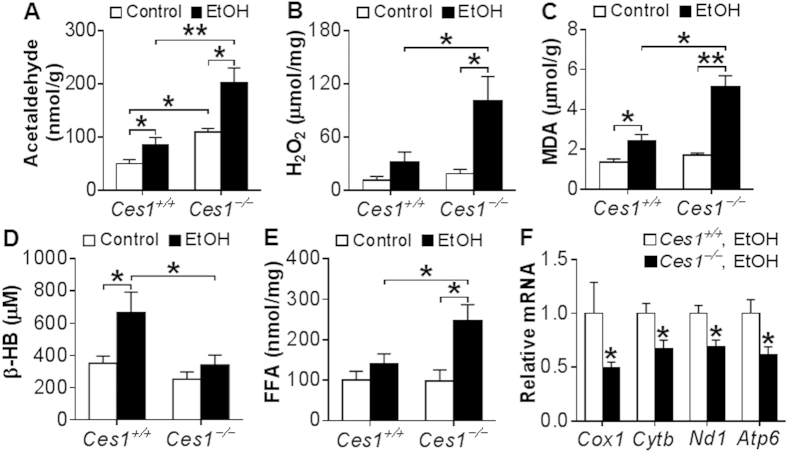
*Ces1*^−/−^ mice have increased hepatic acetaldehyde level, oxidative stress and mitochondrial dysfunction upon alcohol feeding. *Ces1*^+/+^ and *Ces1*^−/−^ mice were subjected to alcohol feeding as described in the legend of Fig. 5. Hepatic acetaldehyde levels were analyzed using HPLC (**A**). Mitochondrial hydrogen peroxide (H_2_O_2_) levels were analyzed using Amplex Red reagent/horseradish peroxidase, and fluorescent activities were determined (**B**). Hepatic level of MDA (**C**) and plasma levels of β-HB (**D**) and FFA (**E**) were determined. Hepatic mRNA levels of mitochondrial genes were quantified (**F**). *p < 0.05, **p < 0.01. Unpaired Student *t*-test was used for statistical analysis.

To understand how CES1 inactivation aggravates ethanol-induced liver injury, we first measured acetaldehyde level in the liver. Acetaldehyde is a reactive compound that is highly toxic to hepatocytes because it can sensitize cells to oxidative stress or other damaging signals, ultimately leading to mitochondrial damage and cell death^2^,^19^. At the basal level, hepatic acetaldehyde level was increased by 2.2 fold in *Ces1*^−/−^ mice in comparison to *Ces1*^+/+^ mice (Fig. 6A). Upon ethanol feeding, hepatic acetaldehyde level was increased by 1.7 fold in *Ces1*^+/+^ mice and 2 fold in *Ces1*^−/−^ mice (Fig. 6A). Hepatic acetaldehyde level in ethanol-fed *Ces1*^−/−^ mice was 2.4 fold higher than in ethanol-fed *Ces1*^+/+^ mice (Fig. 6A). Consistent with these observations, the mRNA level of acetaldehyde dehydrogenase 2 (*Aldh2*) was reduced by 50% in *Ces1*^−/−^ mice (*p* < 0.05; data not shown).

Oxidative stress is an important contributor to the pathogenesis of ALD. Mitochondrial reactive oxygen species (ROS) triggers pro-inflammatory cytokine production, damages mitochondrial DNA and promotes lipid peroxidation^20^,^21^,^22^. We therefore examined whether the aggravated liver inflammation in *Ces1*^−/−^ mice was associated with increased ROS. Indeed, upon alcohol feeding hepatic levels of hydrogen peroxide (H_2_O_2_) (Fig. 6B) and malondialdehyde (MDA) (Fig. 6C) in *Ces1*^−/−^ mice were increased by 5.5 fold and 3 fold, respectively, whereas these increases were less pronounced in *Ces1*^+/+^ mice (Fig. 6B,C). In addition, hepatic H_2_O_2_ and MDA levels were increased by 3 fold and 2 fold, respectively, in ethanol-fed *Ces1*^−/−^ mice than in ethanol-fed *Ces1*^+/+^ mice (Fig. 6B,C), indicating that CES1 is critical for preventing ethanol-induced oxidative stress.

Ethanol feeding significantly increases acetate level and therefore enhances the synthesis of acetyl-CoA and β-hydroxybutyrate (β-HB). On the other hand, elevated mitochondrial FAO is also accompanied by increased β-HB synthesis. As such, plasma β-HB level may reflect mitochondrial functions under normal conditions. As shown in Fig. 6D, ethanol significantly increased plasma β-HB level in *Ces1*^+/+^ mice but not in *Ces1*^−/−^ mice. In contrast, ethanol induced a 2.5-fold increase in hepatic FFA level in *Ces1*^−/−^ mice but not in *Ces1*^+/+^ mice (Fig. 6E). The increase in hepatic FFA level was not accompanied by any change in genes involved in fatty acid synthesis (*Srebp1c*, *Fas*, *Acc1*, *Acc2*), transport (*CD36*, *Fabp1*) or oxidation (*Ppar*α, *Cpt1*α, *Mcad*, *Acox1*, and *Acox2*) (see Supplementary Fig. S2). These data suggest that *Ces1*^−/−^ mice may have an impaired mitochondrial function.

Increased acetaldehyde and ROS production is a crucial promoter of mitochondrial DNA mutation, degradation and mitochondrial dysfunction^20^,^21^,^22^,^23^. We then tested mRNA levels of genes encoding mitochondrial enzymes. *Ces1*^−/−^ mice had a significant reduction in mRNA levels of cytochrome oxidase subunit I (*Cox1*), cytochrome *b* (*Cytb*), NADH dehydrogenase subunit 1 (*Nd1*) and ATP synthase F0 subunit 6 (*Atp6*) (Fig. 6F). Together, the data of Fig. 6 demonstrate that global deletion of CES1 aggravates alcohol-induced acetaldehyde and ROS production and mitochondrial dysfunction.

### Hepatic CES1 deficiency exacerbates alcohol-induced liver steatosis

**Figure 7:**
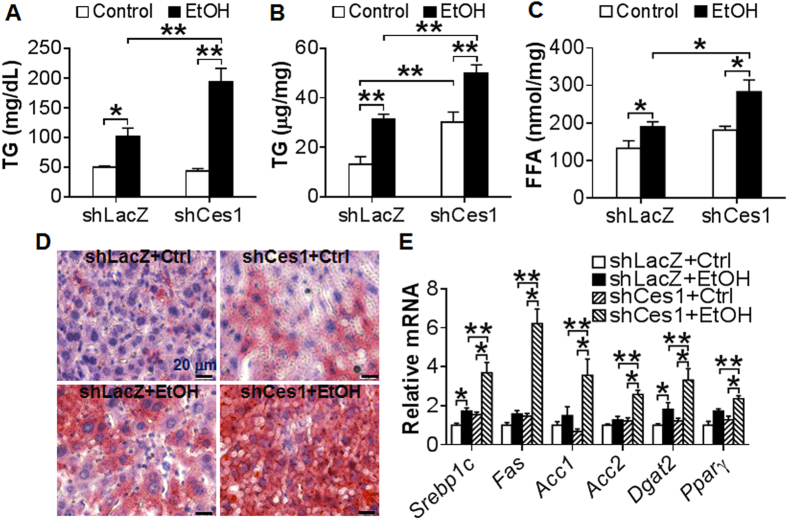
Hepatic CES1 deficiency exacerbates alcohol-induced liver steatosis. (**A**–**E**) C57BL/6J mice were fed a Lieber-DeCarli control liquid diet for 5 days. On the 6^th^ day, mice were injected i.v. with Ad-shLacZ or Ad-shCes1 and then fed either a Lieber-DeCarli ethanol diet or pair-fed a Lieber-DeCarli control diet for 10 days. On the 16^th^ day, mice were gavaged with a single dose of ethanol (5 g/kg) or isocaloric maltose dextrin. Plasma TG (**A**), hepatic TG (**B**) and hepatic FFA (**C**) levels were determined. Representative oil red O stainings were shown (**D**). Hepatic mRNA levels of lipogenic genes were quantified (**E**). *p < 0.05, **p < 0.01. Unpaired Student *t*-test was used for statistical analysis.

In addition to the liver, CES1 is also expressed in other tissues, such as intestine, adipose tissue and macrophages. We therefore investigated whether the aggravated liver injury observed in global *Ces1*^−/−^ mice was due to inactivation of hepatic *Ces1*. Consistent with a role in TG hydrolysis, over-expression of *Ces1* prevented alcohol-induced TG accumulation in AML12 cells (see Supplementary Fig. S3), a murine hepatocyte cell line. We then fed C57BL/6 mice a Lieber-DeCarli control diet for 5 days. On day 6, the mice were fed either a Lieber-DeCarli control diet or 5% ethanol diet and also injected i.v. with adenoviruses expressing *Ces1* shRNA (Ad-shCes1) or LacZ shRNA (Ad-shLacZ; control) as described^12^. On day 16, the mice were gavaged with a single dosage of ethanol (5 g/kg). Mice infected with Ad-shCes1 had a ~97% reduction in hepatic *Ces1* mRNA level (See Supplementary Fig. S4A). Body weight and food intake were comparable between the two genotypes (see Supplementary Fig. S4B and S4C). Alcohol treatment led to increased plasma and hepatic levels of TG in both the *Ces1*-deficient mice and control mice (Fig. 7A,B). In addition, alcohol-fed *Ces1*-deficient mice had ~2 fold increase in plasma or hepatic TG levels than alcohol-fed control mice (Fig. 7A,B). Similar results were observed for hepatic FFAs (Fig. 7C). In contrast, there was no change in hepatic cholesterol levels among the different groups (see Supplementary Fig. S4D). Oil red O staining of liver sections also confirmed hepatic TG accumulation (Fig. 7D). Consistent with the change in hepatic TG levels, knockdown of *Ces1* in ethanol-fed mice markedly increased mRNA levels of lipogenic genes, including *Srebp-1*, *Fas*, *Acc1*, *Acc2*, diacylglycerol O-acyltransferase 2 (*Dgat2*) and peroxisome proliferator-activated receptor γ (*Ppar*γ) (Fig. 7E). Thus, the data of Fig. 7 indicate that hepatic CES1 deficiency aggravates ethanol-induced liver steatosis via inducing lipogenic genes.

### Hepatic CES1 deficiency exacerbates alcohol-induced hepatitis

**Figure 8:**
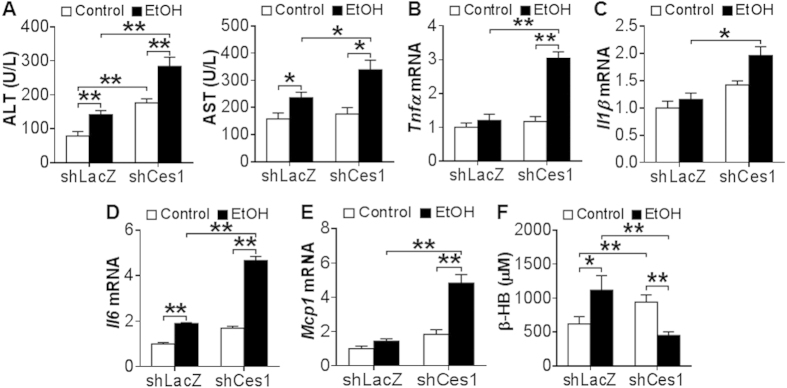
Hepatic CES1 deficiency exacerbates alcohol-induced liver inflammation. (**A**–**F**) Mice were subjected to alcohol feeding as described in the legend of Fig. 7. Plasma levels of ALT (**A**) and AST (**B**) as well as hepatic mRNA levels of *Tnf*α (**C**), *Il1*β (**D**), *Il6* (**E**) and *Mcp-1* (**F**) were determined. Plasma β-HB level was quantified (**G**). *p < 0.05, **p < 0.01. Unpaired Student *t*-test was used for statistical analysis.

Next, we determined whether hepatic CES1 deficiency affected liver injury. Ethanol induced plasma ALT (Fig. 8A) and AST (Fig. 8B) levels in both the control mice and *Ces1*-deficient mice. Hepatic *Ces1* deficiency further increased plasma ALT and AST levels (Fig. 8A,B). In addition, ethanol significantly induced hepatic mRNA levels of *Tnf*α (Fig. 8C), *Il1*β (Fig. 8D), *Il6* (Fig. 8E) and *Mcp1* (Fig. 8F) in *Ces1*-deficient mice but not in the control mice except for *Il6*. Furthermore, hepatic *Tnf*α, *Il1*β, *Il6* and *Mcp1* mRNA levels were increased by >2 fold in ethanol-fed *Ces1*-deficient mice than in ethanol-fed control mice (Fig. 8C–F). Finally, ethanol increased plasma β-HB levels in the control mice but reduced plasma β-HB levels in the *Ces1*-deficient mice (Fig. 8G), suggesting that hepatic CES1 deficiency may cause mitochondrial dysfunction. Collectively, the data of Figs 7 and 8 demonstrate that hepatic CES1 deficiency is sufficient to aggravate alcohol-induced steatohepatitis.

## Discussion

CES1 is a drug-metabolizing enzyme. Its role in liver inflammation or liver injury has not been investigated previously. In the current study, we demonstrate that CES1 protects liver from alcohol- or MCD diet-induced liver inflammation or liver injury. Importantly, we also show that hepatic CES1 is markedly reduced in patients with alcoholic steatohepatitis. Mechanistically, our data suggest that ethanol inhibits CES1 expression likely through reducing HNF4α expression. Thus, the present study suggests that the HNF4α-CES1 pathway may play an important role in the protection against the development of ALD.

CES1 has the ability to hydrolyze amide or ester bonds^24^. Our previous studies show that TG is a substrate for CES1^12^. Defective TG hydrolase activity is responsible for elevated TG accumulation in the liver of *Ces1*-deficient mice. Ethanol is unlikely to be a direct substrate of CES1. However, the metabolites of CES1 may affect alcohol metabolism and mitochondrial functions. When CES1 is deficient, the change in the levels of metabolite(s) and/or substrate(s) of CES1 may cause accumulation of toxic substances, such as acetaldehyde and ROS. These toxic substances are sufficient to cause damages on mitochondria and cell membranes, eventually resulting in liver inflammation and damage. One of our future directions will be to determine which metabolite(s) and substrate(s) cause an increase in these toxic substances. In agreement with our hypothesis, ALDH2 is inhibited in *Ces1*^−/−^mice. A previous report shows that HNF4α also controls ALDH2 transcription^25^, supporting our current finding that the HNF4α-CES1 pathway regulates the development of ALD.

HNF4α is known to control the basal expression of a variety of genes associated with lipid, glucose, bile acid and drug metabolism, differentiation and morphogenesis^26^. The current study identifies CES1, a TG hydrolase in the liver^12^, as a direct target gene of HNF4α, suggesting that HNF4α may also regulate lipolysis. Under the conditions of common metabolic stress (diabetes, obesity and NAFLD, HFD feeding), elevated FFAs, cholesterol, p53 and miR-34a orchestrate to inhibit HNF4α expression^27^. In addition, alcoholism leads to hypozincemia^28^ and zinc deprivation suppresses the DNA-binding activity of HNF4α in HepG2 cells^29^. Thus, ethanol treatment inhibits HNF4α expression and DNA binding activity, likely through inducing lipogenesis and FFA levels and reduced zinc level. It is also possible that other unknown mechanism(s) are involved in inhibiting HNF4α expression.

An unexpected finding in this study is that liver-specific knockdown, but not global inactivation, of CES1 aggravates ethanol-induced liver steatosis. Loss of hepatic CES1 is known to induce lipogenesis and liver steatosis^12^. In addition to the liver, CES1 is also expressed in the intestine, in which CES1 is reported to regulate chylomicron production^30^. We speculate that loss of intestinal CES1 may affect alcohol metabolism and fat absorption, thus resulting in unchanged hepatic TG levels in *Ces1*^−/−^ mice.

Global or liver-specific loss of CES1 causes liver inflammation in the presence or absence of alcohol feeding, underscoring the importance of CES1 in controlling inflammatory response. CES1 deficiency causes elevated levels of acetaldehyde, H_2_O_2_, MDA and FFAs as well as mitochondrial dysfunctions, which are known to contribute to liver inflammation and liver damage. Hepatic FAEE, which is known to cause cytotoxicity, is increased upon alcohol feeding^31^. CES1 is shown to play a role in FAEE metabolism^32^,^33^. Thus, we cannot rule out that FAEE also plays a role in liver damage in *Ces1*^−/−^ mice.

Our recent data show that CES2 is also regulated by HNF4α^34^. CES2 prevents liver steatosis by regulating lipolysis, ER stress and lipogenesis^34^. Similarly, CES1 prevents lipolysis by modulating lipolysis and lipogenesis^12^. These functional similarities between CES1 and CES2 likely result from their triglyceride hydrolase activity. In addition to HNF4α, CES1 is also induced by glucose^14^ and farnesoid X receptor^12^, but repressed during fasting^14^ and alcohol. In contrast, CES2 is repressed in *db*/*db* mice, high fat diet-fed mice and patients with non-alcoholic steatohepatitis. Therefore, the regulation of CES1 and CES2 is largely different. It is unclear whether CES2 also plays a role in ALD.

In summary, the current study demonstrates that CES1 protects liver from ethanol- or MCD diet-induced liver inflammation and damage. The impaired HNF4α-CES1 pathway may contribute to the pathogenesis of ALD. Targeting hepatic CES1 may represent a novel strategy for prevention and treatment of ALD and non-alcoholic liver injury.

## Methods

### Mice, diets and human ALD samples

Liver-specific *Hnf4*α^−/−^ mice were generated by crossing *Hnf4*α^*fl/fl*^ mice with albumin-Cre mice (all from the Jackson Laboratory. Bar Harbor, ME). *Ces1*^−/−^ mice were generated by replacing exon 1 with an ACN-LacZ cassette as described^33^. Detailed characterization of the *Ces1*^−/−^ mice will be reported elsewhere. *Ces1*^−/−^ mice were backcrossed with C57BL/6 mice for at least 6 generations prior to experimentation. MCD diets were purchased from Harlan Laboratories (Cat # TD.90262; Madison, WI). Lieber-DeCarli diets were purchased from Bio-Serv (Flemington, NJ). Human liver samples with alcoholic steatohepatitis (with fibrosis) were obtained from the Liver Tissue Cell Distribution System at University of Minnesota. All the animal experiments were approved by the Institutional Animal Care and Use Committee at Northeast Ohio Medical University (NEOMED). The use of human tissue samples was approved by the Institutional Review Board at NEOMED. All the experiments were performed in accordance with the relevant guidelines and regulations.

### Chronic plus binge alcohol drinking

C57BL/6J mice (12 weeks old) were administered a Lieber-DeCarli control liquid diet (Cat # F1259SP, BioServ) for 5 days. On the 6^th^ day, mice were fed a Lieber-DeCarli liquid diet (Cat # F1258SP, BioServ) containing 5% (vol/vol) ethanol or pair-fed a Lieber-DeCarli control liquid diet for 10 days^18^. On the 16^th^ day, mice were gavaged with a single dose of ethanol (3 or 5 g/kg body weight) or isocaloric maltose dextrin. For some studies, on the 6^th^ day mice were also injected i.v. with either Ad-shLacZ or Ad-shCes1. These adenoviruses have been described previously^12^.

### RNA isolation and quantitative real-time PCR

Total RNA was isolated using TRIzol Reagent (Life Technologies, NY). mRNA levels were determined by quantitative reverse-transcription polymerase chain reaction (qRT-PCR) on a 7500 real-time PCR machine from Applied Biosystems (Foster City, CA). Relative mRNA levels were calculated using the comparative cycle threshold (Ct) method and were normalized to the values of 36B4 mRNA levels.

### Western blotting

Tissues were homogenized in ice-cold modified RIPA buffer and protein concentrations were determined using a Pierce BCA Protein Assay Kit (Thermo Scientific, IL). Antibodies against mouse CES1, HNF4α and β-actin were purchased from Abcam (ab45957, Cambridge, MA), Santa Cruz Biotechnology (sc6556, Santa Cruz, CA) and Novus Biologicals (NB600-501, Littleton, CO), respectively.

### Transient transfection and mutagenesis assays

*Ces1* promoter regions (−1.9 kb, −0.98 kb, −0.3 kb, −0.25 kb or −0.21 kb to +87 bp) were cloned to the pGL3-basic plasmid (Promega, Madison, WI). The mutant pGL3 promoter-luciferase construct [pGL3-Ces1mut(−1.9 k)] was generated using a QuickChange Site-directed Mutagenesis kit from Agilent (Santa Clara, CA). Transient transfection assay was performed as described^35^. pGL3-Ces1 luciferase-reporter constructs were transfected into HepG2 cells using Lipofetamine 3000 (Invitrogen, CA) along with either pcDNA3 or pcDNA3-HNF4α plasmids. After 36 h, luciferase activities were determined and normalized to β-galactosidase activity.

### Chromatin immunoprecipitation (ChIP) assay

ChIP assay was performed using HNF4α antibody following the manufacturer’s instructions (Cat # 17-295, Millipore, MA) as described^17^. qRT-PCR was performed to determine the DNA enrichment. The primer sequences were CAGAACACTGAGGTTTGAATTCC (forward) and TCACACCGACCTAGAGTTTAAAC (reverse), which amplified a fragment between −250 bp and −300 bp in the *Ces1* gene promoter.

### Electrophoretic mobility shift assay (EMSA)

HNF4α protein was generated using a TNT T7 Quick Coupled Transcription/Translation Reactions kit (Cat # L1170, Promega) and pcDNA3-HNF4α plasmid. Oligonucleotides were labeled by biotin on the 3′ end following the manufacturer’s instruction (Cat # 89818, Thermo Fisher Scientific). EMSA was performed using a kit according to the manufacturer’s instructions (Cat # 20148, Thermo Fisher Scientific). The wild-type oligonucleotide sequences used for EMSA were CCCTGTCTGAAGGCCTG CTGTGCTACTCTCTGCCTTTGGGAGGCCGACAG-3′ (top strand) and 5′-CTGTCGGCCTCCCAAAGGCAGAGAGTAGCACAGCAGGCCTTCAGACAGGG-3′ (bottom strand). The mutant oligonucleotide sequences were 5′-CCCTGTCTGAAGGCCTGCTGTGTTACTTTTTGTTTTTGGGAGGCCGACAG-3′ (top strand) and 5′-CTGTCGGCCTCCCAAAAACAAAAAGTAACACAGCAGGCC TCCAGACAGGG-3′ (bottom strand).

### Primary hepatocyte isolation

Mouse primary hepatocytes were isolated as described^36^,^37^. Mice were anaesthetized by intraperitoneal injection of 50 mg/kg pentobarbital. The portal vein was cannulated with a 23-gauge plastic cannula. Mouse livers were perfused with Hank’s Balanced Salt Solution (HBSS, Cat # 14170-112, Thermo Fisher Scientific) containing 0.19 g/L EDTA. Simultaneously, the inferior vena cava was cut open. Subsequently, livers were perfused with HBSS, calcium, magnesium buffer (Cat # 14025092, Thermo Fisher Scientific) with 0.8 mg/mL Collagenase from Clostridium histolyticum type IV (Sigma, St. Louis, MO). Primary hepatocytes were released and collected in a 50 mL centrifuge tube. After centrifugation at 50 × g for 5 minutes and washing with DMEM, cells were cultured in DMEM/10%FBS (Atlanta Biologicals, Georgia, USA) in 6-well plates pre-coated with 0.1% gelatin (Sigma-Aldrich).

### Measurement of hepatic acetaldehyde level using high performance liquid chromatography (HPLC)

Hepatic acetaldehyde level was determined using derivatization with DNPH, followed by HPLC separation as described previously^38^. 100 mg liver tissue was homogenized in 3 M perchloric acid (Fisher Scientific). The pH of the solution was adjusted immediately to 4.0 using 2 volumes 3 M sodium acetate buffer (pH 9.0) (Fisher Scientific). After centrifugation at 12000 rpm at 4 ^o^C for 20 minutes, the supernatant was transferred into an ice-cold tube, followed by addition of 80-fold molar excess 2,4-dinitrophenylhydrazine (DNPH, Cat # 119266, Sigma-Aldrich) in 6 N HCl. The mixture was then placed on a shaker for 1 h at room temperature. Derivatization was stopped with 3 volumes of 3M sodium acetate buffer (pH 9.0). Two volumes of acetonitrile (Sigma-Aldrich) were added to extract AcH-DNP. After centrifugation at 10000 × g for 5 minutes at 4 ^o^C, the organic phase was condensed to 50 μL. The ultra high performance liquid chromatograph (UHPLC) machine was purchased from Shimadzu Corp. (Columbia, MD). A Restek C18 HPLC column (25 cm × 4.6 mm i.d. 5 μm) coupled with an Ultra C18 guard column (10 mm × 4 mm i.d.) were purchased from Fisher Scientific (Pittsburgh, PA). The elution program was described previously^38^. An AcH-DNP standard (Sigma-Aldrich, St.Louis. MO) was used to create a standard curve. The values of the area under the curve of the AcH-DNP peaks were determined to calculate the concentration of acetaldehyde in each sample.

### Malondialdehyde (MDA) assay

MDA assay was performed as described^35^. Buffer I was made by dissolving 2-Thiobarbituric acid (Sigma-Aldrich) in 10% perchloric acid to a final concentration 0.67%. Assay buffer was made by adding 20% trichloroacetic acid to buffer I (2:3,v/v). 1,1,3,3,-tetraethoxypropane (Sigma-Aldrich) was used as a standard. 30 mg liver tissue was homogenized in 500 μL saline. After centrifugation, 100 μL supernatant was collected and added to 1 ml assay buffer. The reaction mixture was incubated at 95 °C for 30 min, cooled down and centrifuged at 3000 rpm for 10 min. The absorbance was measured at OD532 nm.

### Mitochondrial H_2_O_2_ assay

Approximately 50 mg of liver tissue was homogenized in 1 ml of homogenization buffer containing 25 mM Hepes pH7.4, 1 mM EDTA, 0.25 M sucrose, 2 mM MgCl_2_, 1 μM butylated hydroxytoluene (BHT), 1:200 dilution of Sigma P8340 and 1 μM diethylenetriaminepentaacetic acid (Sigma-Aldrich). The homogenates were centrifuged at 500 × g for 5 min to pellet nuclei and debri. The resultant supernatant was centrifuged at 10,000 × g for 10 min at 4 °C to obtain mitochondria. The pellets were resuspended in 150 μl homogenization buffer. About 20 μL solution was saved for measuring protein concentration.

Mitochondrial H_2_O_2_ was detected using Amplex Red assay as described^39^. The working solution was prepared using 100 μL of 10 mM Amplex Red reagent (Thermo Fisher Scientific), 2 μL of 1000 U/ml horseradish peroxidase (HRP) and 10 ml of 50 mM potassium phosphate (pH7.7) with 0.5 mM diethylenetriaminepentaacetic acid (Sigma-Aldrich). 50 μL of sample or standard was pipetted into 96 plates, followed by addition of 50 μl of the Amplex Red reagent/HPR working solution. The reaction mixture was protected from light and incubated at room temperature for 30 min. The fluorescence was measured with excitation in the range of 530–560 nm and emission at 590 nm. Mitochondrial H_2_O_2_ levels were normalized to protein levels.

### Lipid and body fat analysis

Plasma triglycerides and cholesterol were measured using Infinity reagents from Thermo Scientific (Waltham, MA). Plasma and hepatic free fatty acids were measured using a kit from Wako Diagnostics (Richmond, VA). To measure lipids in liver, approximately 100 mg liver tissue was homogenized in methanol and extracted in chloroform/methanol (2:1 v/v). Hepatic triglyceride and cholesterol levels were then quantified. Body fat content was determined by EchoMRI (Houston, TX).

### Plasma alanine aminotransferase (ALT) and aspartate aminotransferase (AST) analysis

Plasma ALT and AST levels were determined using Infinity reagents (Middletown, VA) following the manufacture’s instructions.

### Statistical Analysis

The data were analyzed using unpaired Student *t* test and ANOVA (GraphPad Prisim, CA). All values were expressed as mean ± SEM. Differences were considered statistically significant at *P* < 0.05.

## Additional Information

**How to cite this article**: Xu, J. *et al.* Carboxylesterase 1 Is Regulated by Hepatocyte Nuclear Factor 4a and Protects Against Alcohol- and MCD diet-induced Liver Injury. *Sci. Rep.* **6**, 24277; doi: 10.1038/srep24277 (2016).

## Supplementary Material

Supplementary Information
