# Supplementary material for: Carboxylesterase 1 Is Regulated by Hepatocyte Nuclear Factor 4α and Protects Against Alcohol- and MCD diet-induced Liver Injury
Source: Sci Rep. 2016 Apr 14;6:24277. doi: 10.1038/srep24277 (PMC4831009; doi:10.1038/srep24277)
Supplement: Supplementary Information [file srep24277-s1.pdf]

Supplementary Information

Carboxylesterase 1 Is Regulated by Hepatocyte Nuclear Factor 4α and Protects Against Alcohol- and MCD diet-induced Liver Injury

Jiesi Xu,[1] Yang Xu,[1] Yuanyuan Li,[1] Kavita Jadhav,[1] Min You,[2] Liya Yin[1] and Yanqiao Zhang[1]

[1]Department of Integrative Medical Sciences, Northeast Ohio Medical University, Rootstown, OH 44272, USA

[2]Department of Pharmaceutical Sciences, Northeast Ohio Medical University, Rootstown, OH 44272, USA

Corresponding authors:

Yanqiao Zhang, MD

Department of Integrative Medical Sciences

Northeast Ohio Medical University

Phone: (330) 325-6693

Fax: (330) 325-5978; Email: yzhang@neomed.edu

Liya Yin, MD, PhD

Northeast Ohio Medical University

Email: lyin@neomed.edu

Supplementary Figure 1. Hepatic CES1 expression is absent in *Ces1*$^{-/-}$ mice. Hepatic *Ces1* mRNA (A) and protein (B) levels were determined in *Ces1*$^{+/+}$ mice and *Ces1*$^{-/-}$ mice (n=8).

Supplementary Figure 2. Body weight, plasma or hepatic triglycerides and hepatic mRNA levels in alcohol-fed *Ces1*$^{-/-}$ mice. Mice were fed an alcohol diet as described in the legend of Figure 5 (n=8). Body weight before or after the alcohol diet feeding was determined (A). Plasma TG (B) and hepatic TG (C) levels were quantified. Hepatic mRNA levels were analyzed (D). * $p<0.05$, ** $p<0.01$

Supplementary Figure 3. Over-expression of CES1 prevents alcohol-induced triglyceride accumulation in AML12 cells. AML12 cells were infected with Ad-empty or Ad-Ces1 (n=3). After 48 h, TG level was determined. * $p<0.05$.

Supplementary Figure 4. Body weight, food intake and hepatic cholesterol level in alcohol-fed *Ces1*-deficient mice. C57BL/6J mice were injected i.v. with Ad-shLacZ or Ad-shCes1 and then fed a Liber-DeCarli ethanol diet or control diet as described in the legend of Figure 7 (n=8). Hepatic *Ces1* mRNA level (A), body weight (B), food intake (C) and hepatic cholesterol (D) levels were quantified.

Figure S1

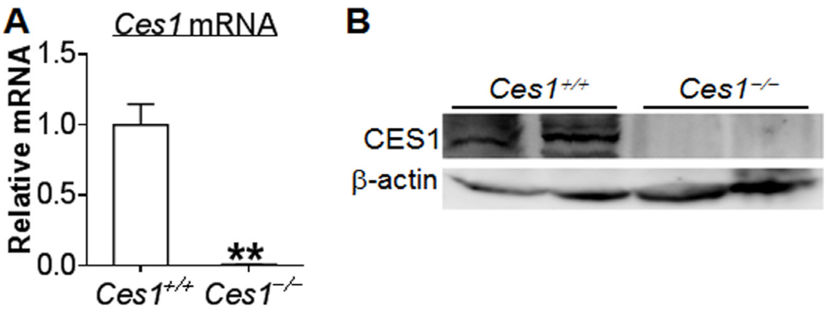
A
Ces1 mRNA
Relative mRNA
1.5
1.0
0.5
0.0
**
Ces1+/+
Ces1−/−
B
Ces1+/+
Ces1−/−
CES1
β-actin

Figure S2

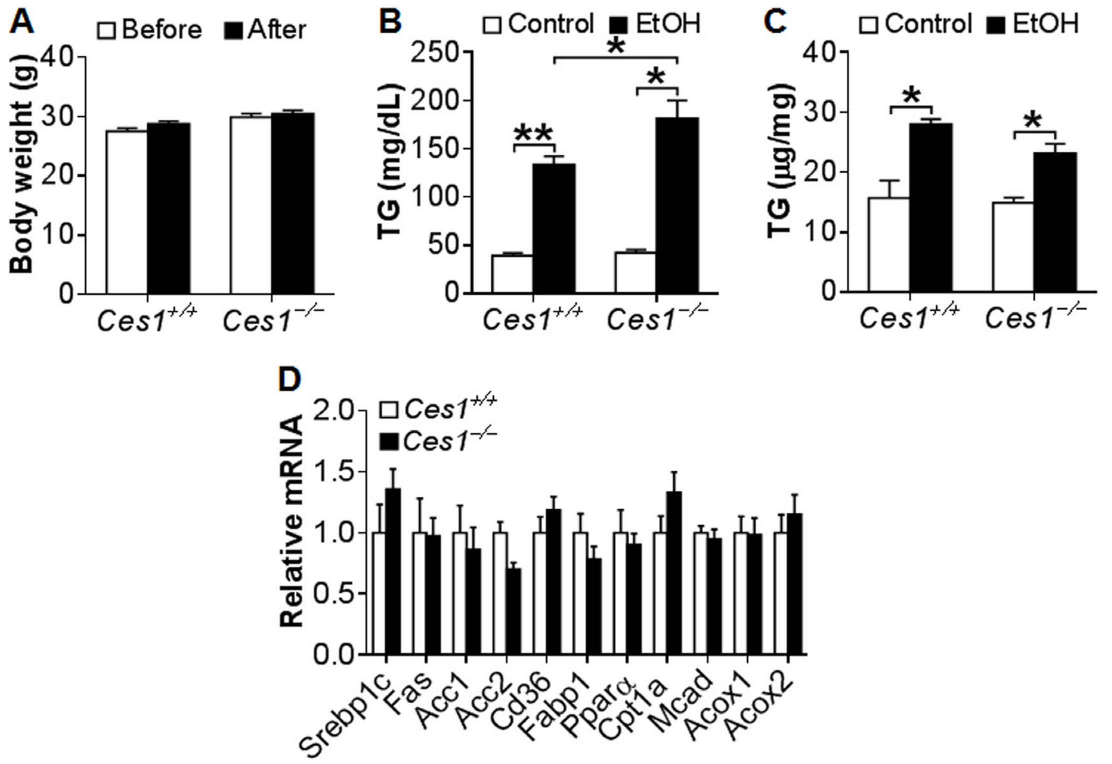
A
Before
After
Body weight (g)
40
30
20
10
0
Ces1+/+
Ces1−/−
B
Control
EtOH
TG (mg/dL)
250
200
150
100
50
0
**
*
*
Ces1+/+
Ces1−/−
C
Control
EtOH
TG (µg/mg)
40
30
20
10
0
*
*
Ces1+/+
Ces1−/−
D
Ces1+/+
Ces1−/−
Relative mRNA
2.0
1.5
1.0
0.5
0.0
Srebp1c
Fas
Acc1
Acc2
Cd36
Fabp1
Pparα
Cpt1a
Mcad
Acox1
Acox2

Figure S3

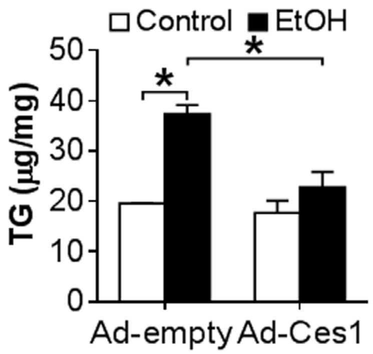

Figure S4

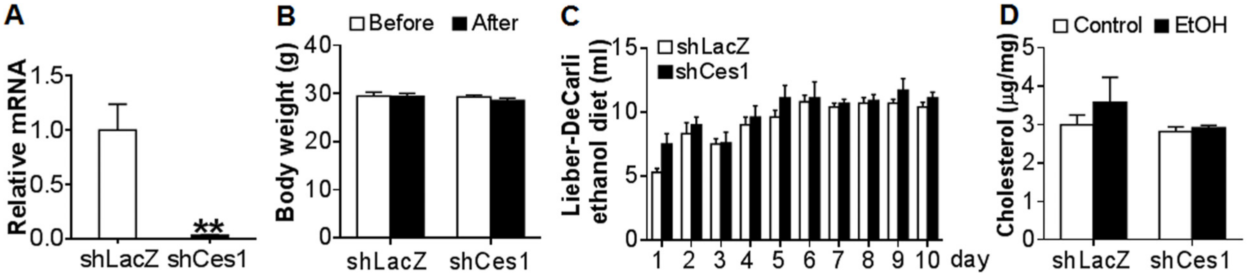
A
Relative mRNA
1.5
1.0
0.5
0.0
**
shLacZ
shCes1
B
Before
After
Body weight (g)
40
30
20
10
0
shLacZ
shCes1
C
Lieber-DeCarli ethanol diet (ml)
shLacZ
shCes1
15
10
5
0
1 2 3 4 5 6 7 8 9 10 day
D
Control
EtOH
Cholesterol (μg/mg)
5
4
3
2
1
0
shLacZ
shCes1
